# Convict cichlid parents that stay with the same mate develop unique and consistent divisions of roles

**DOI:** 10.7717/peerj.10534

**Published:** 2020-12-14

**Authors:** Jennifer L. Snekser, Murray Itzkowitz

**Affiliations:** 1 Department of Animal Behavior, Ecology, and Conservation, Canisius College, Buffalo, NY, United States of America; 2 Department of Biological Sciences, Lehigh Univervsity, Bethlehem, PA, United States of America

**Keywords:** Behavior, Cichlid, Mate fidelity, Repeatability, Parental care, Division of roles

## Abstract

Previous studies, largely on avian species, have suggested that pairs that are permanently monogamous and have biparental care develop a coordination over time that enhances offspring survival. If this is the case, we predicted that a parent involved in biparental care would develop a pattern of biparental care specific to a particular mate and remain consistent in that pattern over time but would lose this pattern if it were to change mates. We tested this prediction with the convict cichlid fish (*Amatitlania nigrofasciata*) which has biparental care that is both complex and flexible. In this species, each parent can perform all parental roles but typically shows a division of labor in which males typically defend against offspring predators while the female typically provides direct care to the offspring. At various times, the parents briefly switch roles. Our experiments revealed that pairs that remained together for two consecutive broods were more consistent in their parental behaviors, including time they spent near the intruder and in the nest compared to pairs that were comprised of individuals that had previously mated with other partners. Also individuals that remained with the same partner were also more consistent as a parental unit, maintaining their sex-specific roles of males defending aggressively against an intruder and females spending more time directly caring for young. While our experiment clearly support our prediction that individuals do develop unique coordination with specific individuals, convict cichlids in nature appear to be largely serially monogamous in which they mate only once before changing partners. Thus, it is likely this coordination may be available in many species that have biparental care but become adaptive when repeated matings become common.

## Introduction

In some species, securing offspring with sufficient food and protecting them against predators is better done by two parents than just one and, therefore, biparental care is presumed to have evolved, and is the predominant strategy of birds and common in cichlid fishes (Gross & Sargent, 1985; Clutton-Brock, 1991; Sefc, 2011). While many studies on biparental care illustrate that two parents are usually better than one, offspring survival has also been shown to increase as the two parents age (e.g., Stearns, 1992; Paitz et al., 2007; Dearborn, Anders & Juola, 2008), at least to a point (Reid et al., 2010; Torres, Drummond & Velando, 2011). There are a variety of ecological and evolutionary hypotheses as to why older parents are more successful than younger parents, including the possibility that as parents become more experienced, they are more effective in providing the necessary care to young (Cézilly & Nager, 1996). Experienced parents may also be better able to integrate their own parental behavior with that of their partner.

It seems quite possible that an individual in a long-term pair bond would modify their behavior in response to the behavior of its partner. Early theoretical models predicted that individuals should compensate at least partially for any change in their partner’s effort (Houston & Davies, 1985; McNamara, Gasson & Houston, 1999; Johnstone & Hinde, 2006), and empirical evidence suggests that partial compensation does occur, at least in birds (Whittingham, Dunn & Robertson, 1994; Saino & Møller, 1995; Harrison et al., 2009), though there are exceptions to this in other species (e.g., Itzkowitz, Santangelo & Richter, 2001; Snekser & Itzkowitz, 2014). But these, often minor, adjustments in parental behavior are frequently observed short-term, within a single breeding bout, as one parent changes behavior and the other parent adjusts. Most biparental species mate multiple times throughout their life, with the same or with different partners.

When examining behavior over multiple broods, do parents change their behavior? House sparrows (*Passer domesticus*) remained consistent in their provisioning to chicks from one brood to the next, both within the same breeding season or across years (Schwagmeyer & Mock, 2003). There are apparent sex differences in the repeatability of parental behaviors from brood to brood, for house sparrows (Schwagmeyer & Mock, 2003; Dor & Lotem, 2010) and for pied flycatchers (*Ficedula hypoleuca*) (Potti, Moreno & Merino, 1999). Surprisingly, consistency in parental behavior across multiple broods has not been well examined, despite extensive focus on behavioral consistency in recent decades (Bell, Hankison & Laskowski, 2009).

Here we examine if biparental convict cichlid (*Amatitlania nigrofasciata*) parents are consistent in their parental behavior over successive broods. This study is the second part of a study that tested if pairs that successfully raised offspring were more likely to form a pair bond and raise offspring compared to individuals that had not previously raised a brood together. The results were clear: there was no benefit in producing a second clutch of offspring with the same partner as compared to mating with a new partner (Snekser & Itzkowitz, 2019). These results are valuable because they allow us to ask if pairs with familiar partners are behaving differently from pairs that have not previously bred with each other, in spite of both groups having similar reproductive success.

Although some species of cichlids pairs do mate repeatedly (Morley & Balshine, 2002) convict cichlids do not often mate successively with the same individual in nature (Wisenden, 1995). However, they do serve well as a non-avian animal model to test the above hypothesis. First, they have an elaborate biparental division of labor when raising their offspring in which the two parents do perform all roles but emphasize different ones. For example, both parents do take direct care of the offspring at all stages of development with the female emphasizing direct offspring care while the male emphasizes defense against offspring predators (e.g., Wisenden, 1995; Snekser & Itzkowitz, 2009). Second, pairs will mate repeatedly within the same tank, successfully raising offspring, in spite of having the availability of alternative mates (M. Itzkowitz, 2008, pers. obs). Third, cichlids have a relatively short reproductive cycle, lasting perhaps 6 weeks, which makes them especially amenable to laboratory experiments and an important part of our experiment is pairs being able to mate again quickly and successfully with a new partner. Finally, a previous laboratory study on this species showed that pairs that mated repeatedly had an increased offspring survival and correspondingly changed their behavior toward their offspring, their mate, and other individuals within the tank (Colgan & Salmon, 1986). Specifically, with successive breeding bouts, females spent increasingly more time in the nest while males focused more on aggressive behaviors. Unlike Colgan & Salmon (1986), our previous study (Snekser & Itzkowitz, 2019) did not indicate an increase in brood size nor any overall parental behavioral differences across breeding bouts. Across both studies, there were very few meaningful differences in behavior when comparing parents that paired with the same versus a new partner (Colgan & Salmon, 1986; Snekser & Itzkowitz, 2019). This is in contrast to studies of avian species, in which mate fidelity has repeatedly been shown to increase reproductive success (e.g., Hatch & Westneat, 2007; Leach et al., 2020), decrease time between broods (e.g., Lifjeld & Slagsvold, 1988), increase adult survivorship (Leach et al., 2020), and strengthen coordination within pairs (e.g., Choudhury, 1995; Ihle, Kempenaers & Forstmeier, 2015).

Here, we utilize the convict cichlid model to explore the consistency in parental behavior specifically focusing on pairs that remain together compared to those with new partners (of equivalent breeding experience). Based on the previous work in cichlids and birds, we hypothesize that individuals will be consistent in their behavior from one brood to the next, especially looking at parental coordination. Within convict cichlids, the division of parental roles typically leads to a sex-specific pattern of behavior in which females provide direct parental care and males are aggressive towards potential brood predators. Our aim is to determine if this sex-typical coordination is consistent from one bout to the next for parents, regardless of their experience with their partner. If behavior is repeatable across broods, this would indicate that parents establish their unique behavioral patterns within their pairings and continue to exhibit those behaviors. We seek to determine if mate fidelity or sex of the parent has an effect on the consistency of parental behavior.

## Materials & Method

Convict cichlids are endemic to many freshwater environments in Central America. Their reproduction begins when males and females pair-off and establish jointly defended territories. Eggs are deposited in small crevices with both sexes fanning the eggs and defending against all offspring predators. Although a number of different species will eat the eggs, the most common predators are other convict cichlids. After about 3 days, the eggs hatch into non-mobile wrigglers (larvae). During this stage, both parents carry all of the wrigglers to a small pre-dug pit. After approximately 6 days, the wrigglers mature into small free-swimming fry that then move about in an amorphous cloud while both parents continue to defend against predators. In all offspring stages, the larger male spends most of his time defending the territory while the female spends most of her time in close contact of her offspring. From time to time, the male will exchange places with the female for a short time period while the female moves about the territory. For more detail on the natural behavior of the convict cichlid see: Wisenden (1994), Wisenden (1995), Wisenden et al. (2008) and Cleveland-Roberts & Itzkowitz (2009).

Lehigh University Institutional Animal Care and Use Committee provided full approval for this research (Animal Welfare Assurance No. A3877). In our laboratory, all convict cichlid fish were kept on a 12:12 L:D photoperiod at a constant temperature (20 ± 2 °C) and fed pellet food *ad libitum* each day. Fish were chosen from laboratory stock populations that were derived from previous laboratory populations, pet suppliers, and wild-caught fish from Costa Rica. Fish used within this experiment had no known previous breeding experience prior to the first arranged breeding bout.

The experimental design was previously described in Snekser & Itzkowitz (2019). Specifically, to produce parental pairs, one male and one female convict cichlid were placed in a 284 L test aquaria. Fish length and mass were measured and recorded. Pairs were arranged based upon size with male fish approximately 10 mm standard length (SL) (rostrum to caudal peduncle) longer than female fish, as is typical in natural populations (Wisenden, 1995).

Each test aquarium contained a clay flower pot that was used as a breeding site and nest. Females oviposit on the inside walls of the pot and young stay within the pot during the egg and wriggler stages. At the end of the tank opposite the nest, a 15 cm ‘intruder area’ was partitioned from the remainder of the tank by using clear Plexiglas (see Itzkowitz, Santangelo & Richter, 2001). Prior to spawning, a small (less than 30 mm SL) conspecific intruder was placed in this area, which is thought to increase pair bonding (Itzkowitz & Draud, 1992). After spawning occurred, the small intruder was removed.

For the first breeding bout, fish were given up to one month to form a pair bond and spawn. Those pairs that do not lay eggs within a month’s time were returned to their original stock tanks. The number of days to spawning and the number of wrigglers that hatched were recorded for each pair (previously reported, Snekser & Itzkowitz, 2019). Behavioral assays began 24 h after the eggs hatched. A convict cichlid intruder approximately 10 mm SL larger than the male of the parental pair was haphazardly chosen from a large stock tank of male convict cichlids and was added to the intruder area. Adult convict cichlids are a primary predator of young convict cichlids (Wisenden, 1994). The intruder was added 5 min before testing began and was removed immediately following testing. All wrigglers were removed from the nest using a 10 mL pipette and counted. 50 wrigglers were placed in the center of the tank, equidistant between the nest and the intruder area, against the front glass. The remaining wrigglers were returned to the nest. Within in this experiment pairs laid an average of 148 (± 9.7 S.E.) eggs, thus the displacement of 50 wrigglers from the nest represented approximately one-third of the offspring.

After wriggler displacement, pairs were video recorded for one hour. Immediately following testing, pairs were removed from the test tank and placed into a new test tank with an opaque black Plexiglas partition in the center of the tank, dividing it in half. The breeding pair was split, with one adult placed on either side of the black partition. After a two-day visual separation period, the black partition was lifted and a clay pot and clear intruder area partition were placed in a set-up identical to the previous breeding bout.

As in Snekser & Itzkowitz (2019), for this second breeding bout, two groups were examined: those that remained with the same partner (*same*) and those that were then paired with a new partner, whose behavior had also been previously recorded in their first breeding bout (*new*) (*N* = 9 for each group). After being placed in the new tank, pairs were given up to 6 weeks to form pair bonds and spawn. Again, the number of days to spawning and the number of wrigglers that hatched from eggs were recorded for each pair. Additionally, if these new-formed pairs failed to successfully spawn, the reason for lack of spawning (death of either parent, eggs eaten, or took longer than 6 weeks) was recorded (see Snekser & Itzkowitz, 2019). Following hatching, testing occurred as during the initial breeding bout.

For video recording during each of the two breeding bouts, fish were left undisturbed and digitally recorded for 1 h following the addition of the intruder and the displacement of 50 wrigglers. Behaviors were scored from DVDs using JWatcher behavioral event recorder software (UCLA & Macquarie University) from the time of placement of wrigglers away from the nest until the last wriggler had been retrieved. This final retrieval was determined by visually confirming that all wrigglers were retrieved when the video camera was turned off and then watching the entire 1-hour video. Only the behavior exhibited by the parents during the retrieval period was analyzed, as parental behaviors are most robust during the period in which young are displaced and vulnerable. All pairs retrieved the displaced wrigglers within the one-hour period.

Parental behaviors recorded included retrievals, total time spent collecting, time in the nest, time with the intruder, and aggressive behaviors toward the intruder. “Retrievals” were defined as the number of times each parent ‘picked up’ displaced wrigglers with its mouth. Though 50 wrigglers were displaced for each pair, the number of retrievals varied, as some parents pick up more than one wriggler at a time, while others repeatedly mouth at the wrigglers, perhaps dropping them before picking them back up, and then return to the nest. The “total time spent collecting” is the number of seconds from the times of displacement of the altricial wrigglers until the final wriggler was placed back within the nest. “Time within the nest” was calculated as the total time each parent spent within the nest (pot) and then divided by the total time that it took parents to retrieve all the young. “Time with intruder” included the amount of time that the fish spent within two body lengths of the intruder, again divided by the total time spent retrieving all displaced wrigglers. The rate of aggression behaviors was calculated by the number of bites and lateral displays divided by minutes near the intruder. Additionally, parental behaviors were calculated by considering the overall result for the intact pair. Data were collected as previously described in Snekser & Itzkowitz (2019). Specifically, the data for male and female cichlids with new partners are presented separately, but are still a representation of the intact pair that the individual was part of and are not the behaviors of a sole individual. For pairs of parents, calculations were as follows: the total number of retrievals by both parents, the overall rate of aggression for both parents, and the time that at least one parent was either near the intruder or within the nest. Additionally, the proportion of time spent together at the intruder and the proportion of time that parents spent displaying strict role division (i.e., female near nest, male near intruder) were calculated.

Statistics were performed with SPSS Version 26. Normality of count data was confirmed by the One-Sample Kolmogorov–Smirnov test (*p* > 0.05) and proportional data was Arcsine transformed (radians) for analysis. Consistency across the two breeding bouts was measured by calculating Repeatability by means of the Intraclass Correlation Coefficient (ICC) with a Two-Way Mixed Model with Absolute Agreement found within the inter-trial reliability measure in SPSS (Lessels & Boag, 1987; Baker et al., 2018). ICCs are reported with 95% confidence intervals (Wolak, Fairbairn & Paulsen, 2012). The two experimental groups (remain with the same partner / new partner) and the behaviors of the two sexes (male/female) were analyzed separately to obtain ICC measures for all dependent variables.

Repeatability is a measure of consistency that has been repurposed from quantitative genetics studies of hereditability and represents a standardized metric that allows comparisons of different behavioral traits and across studies (Bell, Hankison & Laskowski, 2009; Wolak, Fairbairn & Paulsen, 2012; Nakagawa & Schielzeth 2010). Repeatability values can be directly compared, with higher scores (closer to 1.0) representing higher levels of consistency in behavior. Here, we consider Repeatability (R) scores higher than 0.4 to be moderately repeatable and scores above 0.7 to be highly repeatable (*p* < 0.05) (adapted from Baker et al., 2018).

## Results

**Figure 1:**
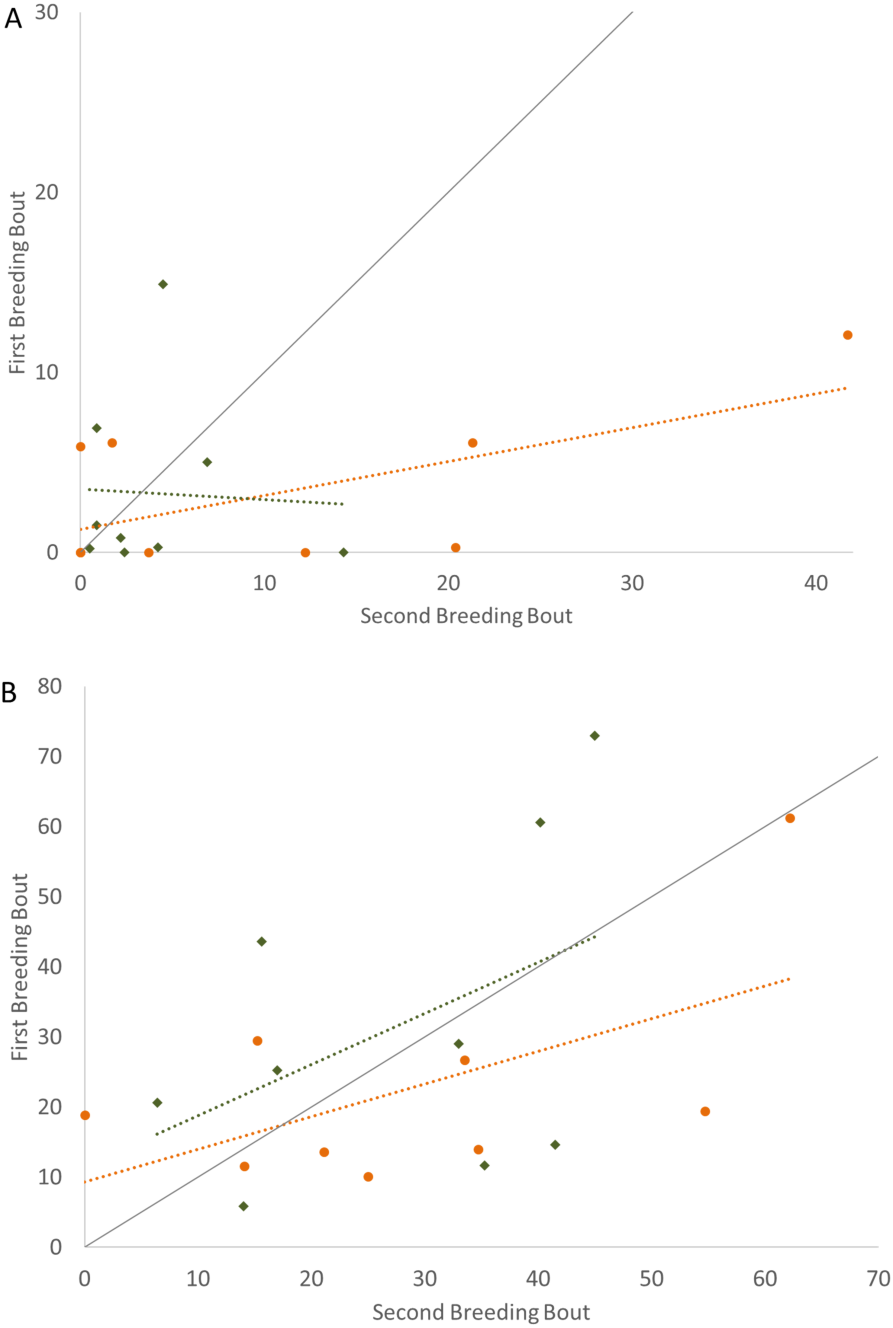
Time spent in the nest with altricial young (percent of total time observed) during the first and second breeding bouts. Parental behavior exhibited by male (A) and female (B) convict cichlids that remained with the same partner (orange circles) or mated with a new partner (green diamonds). Dotted colored lines represent linear regressions for each experimental group. Diagonal gray line represents perfect Repeatability (*R* = 1) between breeding bouts.

**Figure 2:**
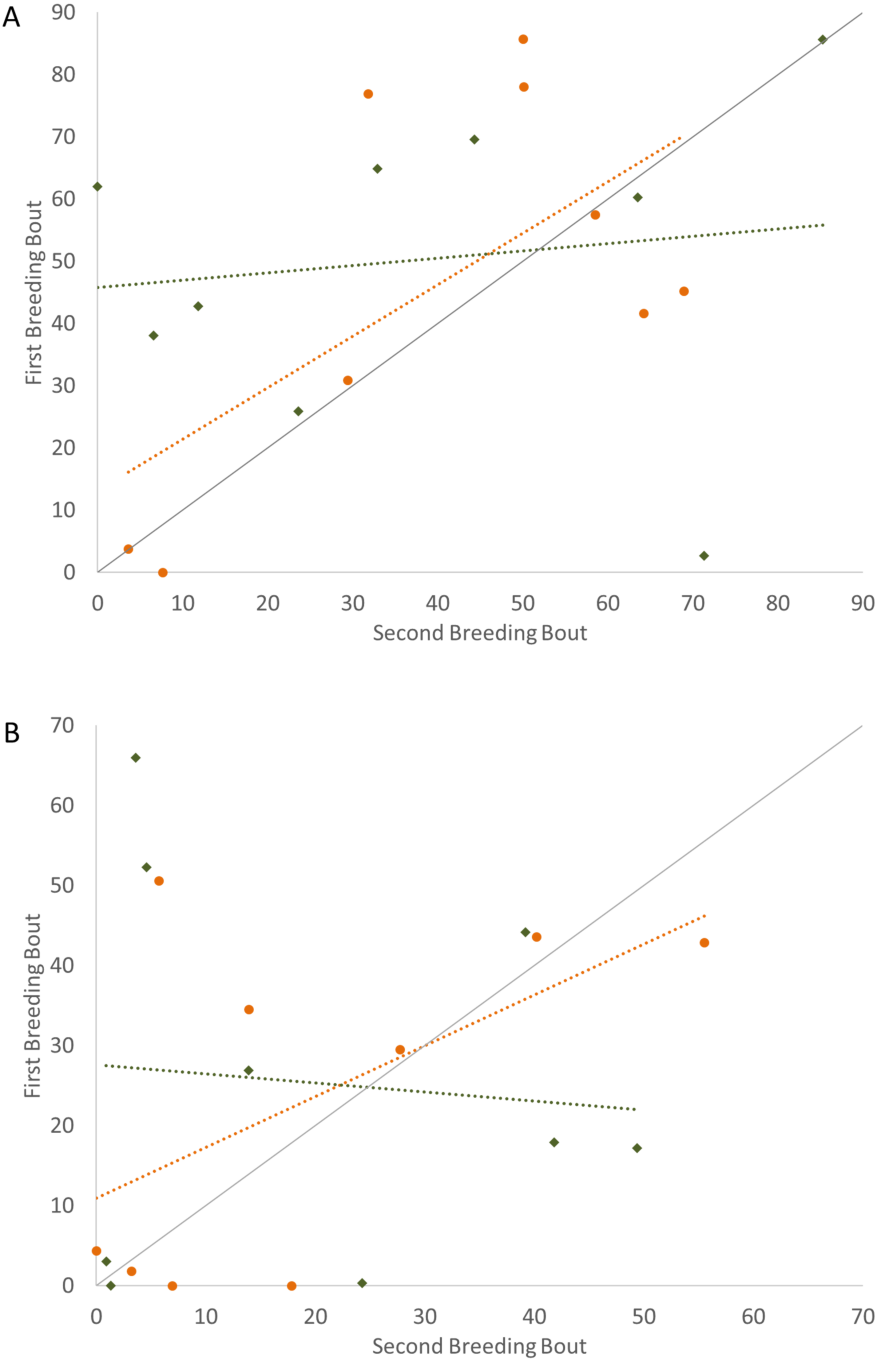
Time spent engaged in defense against a conspecific intruder (percent of total time observed) during the first and second breeding bouts. Parental behavior exhibited by male (A) and female (B) convict cichlids that remained with the same partner (orange circles) or mated with a new partner (green diamonds). Dotted colored lines represent linear regressions for each experimental group. Diagonal gray line represents perfect Repeatability (*R* = 1) between breeding bouts.

In examining the consistency in parental care for male and female convict cichlids that either re-mated with the same partner or with a new partner, significant repeatability was most apparent in the behaviors of those individuals that remained together for a second breeding bout. For these parents that remained with their partners, we found that the proportion of time that parental cichlids spent in the nest (Fig. 1; Table 1) and the proportion of time that each parent spent with a brood predator (Fig. 2; Table 1) were highly repeatable. The number of retrievals was not seen to be consistent, indicating that both male and female parents varied in the number of young that the individual retrieved for each brood. When male or female fish produced a second brood with a new partner, the four parental behaviors measured were not found to have high repeatability. The one exception to this was the amount of time spent within the nest with wrigglers by female convict cichlids. When re-mated with a new male, females showed moderate levels of repeatability, with the tendency to spend more time within the nest during the second breeding bout (Fig. 1B; Table 1). Generally, convict cichlid female parents spend significantly more time within the nest compared to males, regardless of experimental condition (Snekser & Itzkowitz, 2009; Snekser & Itzkowitz, 2014; Snekser & Itzkowitz, 2019).

When considering the pair as a behavioral unit, a similar pattern is apparent: significant and moderate levels of repeatability are exhibited most often by pairs that remain with the same partner. Higher Repeatability scores are seen for the proportion of time spent within the nest, the rate of aggression, and the proportion of time spent with the intruder. The only exception to the trend that pairs remaining together show more repeatability is the measure of the number of retrievals (Table 2).

**Table 1:** Repeatability scores, 95% Confidence Intervals, and *P*-values for various measures of individual parental care across two breeding bouts for males and females that remained together or that mated with a New Partner (of similar experience). Bold values indicate statistically significant and italics represent moderate and high Repeatability scores.

	Pair remains together				New Partner			
		*R*	95% CI	*P*		*R*	95% CI	*P*
% time in nest	Male	*0.544*	−.338–.814	0.144	Male	−0.173	−.678–.581	0.587
	Female	*0.599*	−.777–.910	0.109	Female	*0.563*	−.938–.901	0.132
Retrievals	Male	0.096	−.601–.661	0.445	Male	0.265	−2.26–.834	0.337
	Female	0.245	−2.35–.830	0.351	Female	0.029	−3.30–.781	0.484
% time near intruder	Male	***0.802***	.122–.955	0.017	Male	0.100	−2.99–.797	0.443
	Female	*0.681*	−.415–.928	0.063	Female	−0.148	−2.78–.808	0.413
Rate of Aggression	Male	*0.588*	−.827–.907	0.116	Male	*0.555*	−.973–.900	0.137
	Female	*0.428*	−1.53–.871	0.223	Female	0.247	−2.34–.830	0.349

**Figure 3:**
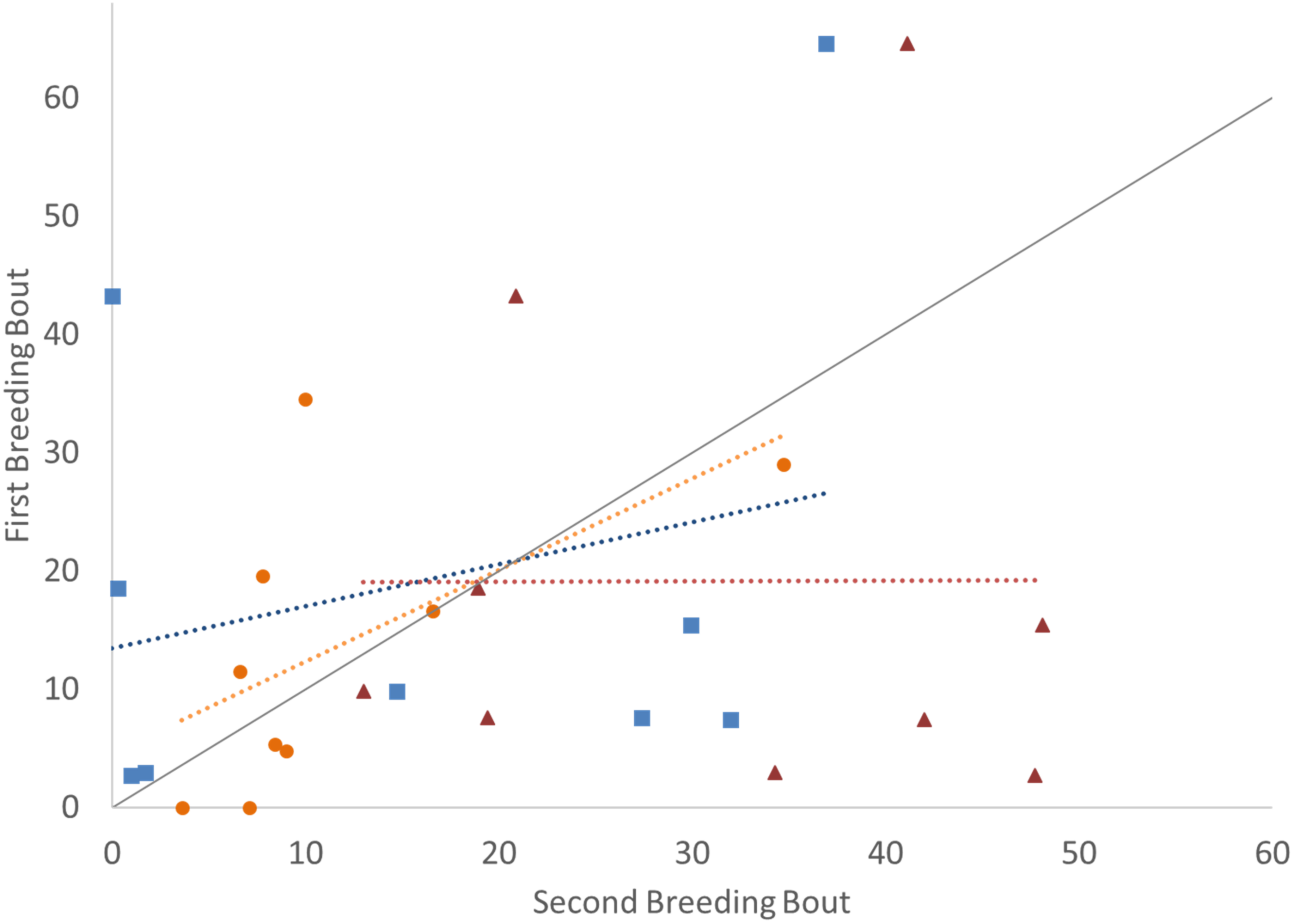
Time that the pair spent divided by parental roles (male with intruder; female in nest) (percent of total time observed) during the first and second breeding bouts. Parental coordination of convict cichlids that remained with the same partner (orange circles) or mated with a new partner (male with new partner = blue squares; female with new partner = red triangles). Dotted colored lines represent linear regressions for each experimental group. Diagonal gray line represents perfect Repeatability (*R* = 1) between breeding bouts.

**Figure 4:**
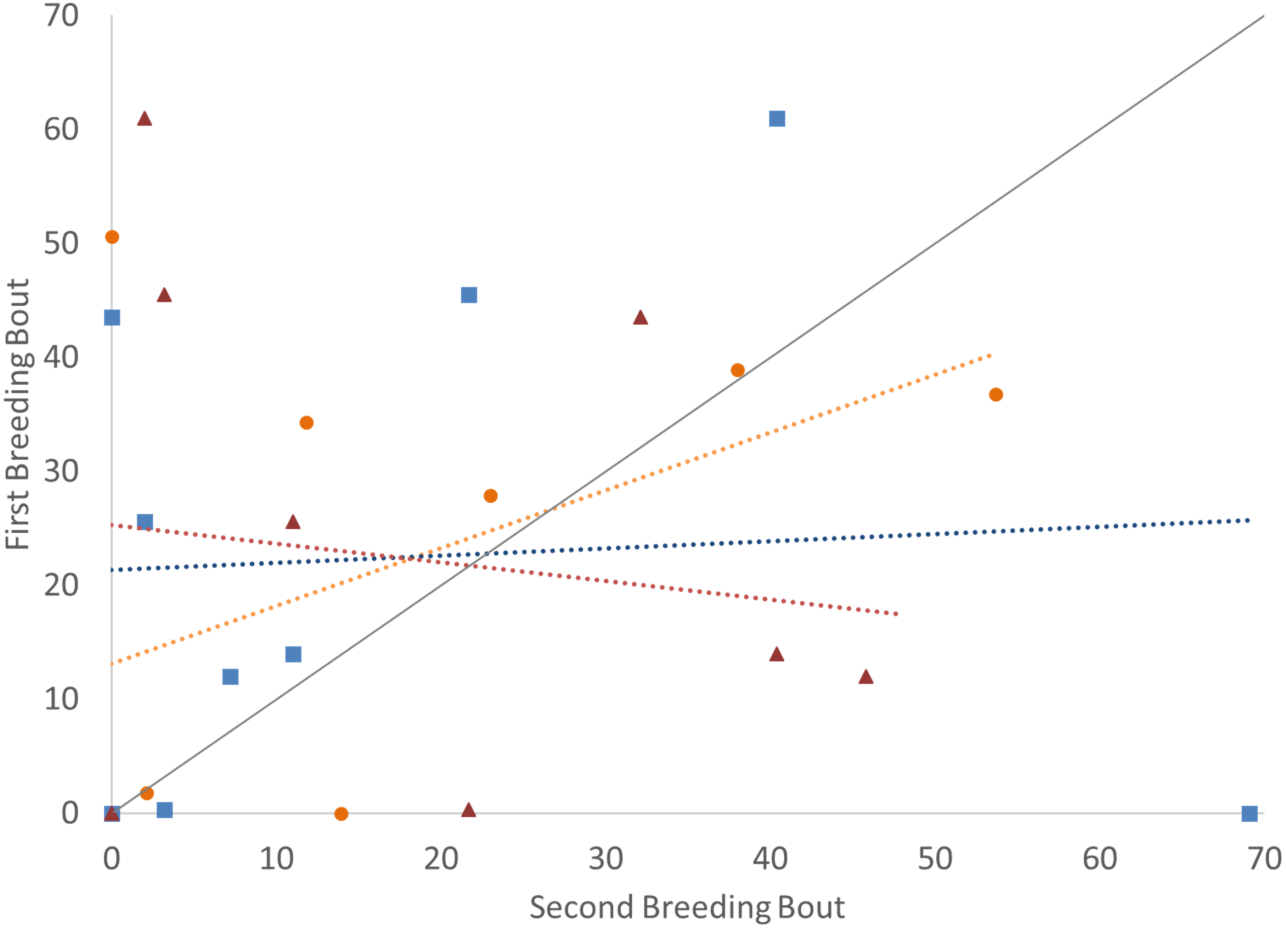
Time that the pair spent together engaged in defense against a conspecific intruder (percent of total time observed) during the first and second breeding bouts. Parental coordination of convict cichlids that remained with the same partner (orange circles) or mated with a new partner (male with new partner = blue squares; female with new partner = red triangles). Dotted colored lines represent linear regressions for each experimental group. Diagonal gray line represents perfect Repeatability (*R* = 1) between breeding bouts.

To determine if the parental unit functioned similarly from one breeding bout to the next, the typical division of roles was also considered, examining the time the parents split activities, with parents engaged within their sex-typical roles: males engaged in aggressive behaviors with the intruder and females at the nest with the offspring. Again, it was only pairs that remained together for both breeding bouts that exhibited high Repeatability scores (Fig. 3; Table 2). Male and female convict cichlids that paired with new mates for their second breeding bout were not consistent in their strict division of parental roles (male at intruder and female with offspring) (Fig. 3; Table 2). The proportion of time that parents spent together engaged in aggressive behaviors with the intruder was also considered, and, again, parents with new partners did not show consistent behaviors, while re-mated partners that stayed together showed moderate Repeatability in behavior (Fig. 4; Table 2).

## Discussion

A presumed benefit of long-term monogamy with biparental care is the information parents gain by mating repeatedly with the same partner; this, in turn, is predicted to increase offspring survival (e.g., Choudhury, 1995; Hatch & Westneat, 2007; Leach et al., 2020). We tested the effects of repeated matings with the same partners using convict cichlid fish and, based on the previous work in cichlids and birds, we hypothesized that parents would be consistent in their behavior with moderate to high levels of repeatability from one brood to the next. We also sought to determine if repeatability was influenced by mate fidelity or the sex of the parent. Parental behavior in convict cichlids is both complex and flexible, with male and female parents adjusting behavior based on the behavior of their partners (Itzkowitz, Santangelo & Richter, 2001; Itzkowitz, Santangelo & Richter, 2002; Snekser & Itzkowitz, 2014). Given this flexibility, we reasoned that parents might show individual differences in the care they provide which would be integrated into their partner’s corresponding behavior to successfully rear the offspring, especially when raising a brood with a different mate.

**Table 2:** Repeatability scores, 95% Confidence Intervals, and *P*-values for various measures of parental care across two breeding bouts for pairs of fish that remained together and for pairs that mated with a New Partner (of similar experience). For each behavior, the pair is considered as a single parental unit and therefore Repeatability scores represent the consistency of the overall behavior of the pair. Bold values indicate statistically significant and italics represent moderate and high Repeatability scores.

	Pair remains together				New Partner		
	*R*	95% CI	*P*		*R*	95% CI	*P*
% time in nest	*0.560*	−.951–.901	0.133	Male	−1.438	−9.81–.450	0.885
				Female	0.349	−1.88–.853	0.279
Retrievals	***0.773***	−.007–.949	0.026	Male	***0.824***	.219–.960	0.012
				Female	*0.694*	−.356–.931	0.057
% time near intruder	***0.788***	.060–.952	0.021	Male	0.123	−2.87–.802	0.428
				Female	0.291	−2.14–.840	0.319
Rate of Aggression	*0.507*	−1.19–.889	0.169	Male	−0.681	−.899–.022	0.972
				Female	−0.136	−.669–.592	0.569
Total time spent retrieving	−0.413	−1.00–.387	0.841	Male	0.186	−.475–.728	0.301
				Female	0.063	−.555–.661	0.429
% time divided by role	*0.691*	−.371–.930	0.059	Male	0.279	−2.20–.837	0.327
				Female	*0.520*	−1.13–.892	0.160
% time together at intruder	*0.659*	−.512–.923	0.075	Male	0.055	−3.19–.787	0.469
				Female	0.276	−2.21–.837	0.329

We examined changes in behavior at both the individual and the pair level. At the individual level, consideration of Repeatability scores (see Methods for detailed explanation of Repeatability scores) for all of the parental behaviors measured indicate that male and female parents that remain together do show a greater degree of repeatability than both male and female convict cichlids that were with new mates for their second brood, for the most of the parental behaviors examined. Overall, these data support the hypothesis that convict cichlids show significant repeatability in certain parental behaviors across breeding bouts, but only when they remain with their same partner.

We further investigated this repeatability of behavior by examining the combined activity of both parents. This is an important perspective to consider, as it is possible that individual parents may alter their behavior from brood to brood, but the couple may still maintain consistent levels of parental contributions. For example, perhaps when an intruder is present, all pairs, irrespective of their experience together, will have at least one member confronting the intruder. In this case, individuals may vary their time but the total time a pair spends engaged in defense may show repeatability. This possibility was not fully supported. When considered as a combined parental unit, again, pairs with the same partners showed high repeatability for time they spent near the intruder. Compared to pairs that were made up of newly coupled partners, pairs that remained together reliably showed moderate to high levels of Repeatability for nearly all of the different parental behaviors measured. An exception to this finding of consistency for pairs that remain together appears when we examine the total time spent retrieving the displaced offspring. A closer look at this measure reveals that pairs that remained together seemed to be (on average) slower at retrieving young in the second bout. Pairs comprised of new partners were generally faster during the second bout (though no significant difference was found between the groups; Snekser & Itzkowitz, 2019). This decrease/increase in the behavior from bout to bout would explain the lack of absolute consistency for time spent retrieving. Additionally, when examining the pair, all pairs showed high Repeatability, most likely due to the experimental design in which all pairs were forced to retrieve the same number (50) of displaced young. Despite these exceptions, most other direct and indirect parental behaviors were generally consistent for pairs that remained together but not for pairs in which parents were newly partnered.

Further, it appears that pairs that remain together are also more consistent in their coordination. Unlike many other biparental species that have a division of labor, convict cichlids parents display a division of parental roles (Smith-Grayton & Keenleyside, 1978; Itzkowitz, 1984; Keenleyside, 1985; Wisenden, 1995) and are capable of performing separate but complimentary responsibilities. We predicted that prior mating experience with the partner would allow the two parents to develop a unique partitioning of their roles, either by spending more time synchronously performing an activity or performing their alternate roles. The time that both parents spent together at the intruder was moderately repeatable when pairs remained together. Further, the pairs’ division of the two basic roles (female spending time at offspring and male spending his time at the intruder) was moderately Repeatable indicating that familiar pairs divided their roles consistently. Those pairs with new partners (either a male or a female) showed far less Repeatability from one brood to the next. They did not consistently coordinate their individual parental contributions.

The fact that some level of Repeatability exists for most of the parental behaviors examined (when partners remain together) is supportive of the hypothesis that mate fidelity results in greater consistency in parental behaviors, but it is unclear why time spent engaged with the intruder offered the most consistent behaviors. We offer two possibilities: first, it is possible that these other activities are different in importance and only the time near a threatening offspring predator has the importance for parents to more precisely adjust their activities. Second, our measurements of these other activities may be inaccurate. For example, time in the nest, performing a combination of aggressive activities were based on time while offspring retirieval was not based on time but rather on the number of times performed. Perhaps the unit of activities should be based on a more general component that could be applied to all activities. .

Overall, our results support the hypothesis that individuals within long-term pair bonds do maintain their biparental behavior across breeding bouts and this is different from other pairs comprised of new partners. Males and females were similar in the levels of repeatability, both when with a new or with the same partner. Little evidence exists to support the hypothesis that behavioral consistency of parents also enhances offspring survival in this fish, as it does in birds (Bradley et al., 1990; Dubois & Cézilly, 2002; Adkins-Regan & Tomaszycki, 2007). Previous studies with the convict cichlid indicate that mate fidelity has little influence on offspring survival (Colgan & Salmon, 1986) and on brood size (Colgan & Salmon, 1986; Snekser & Itzkowitz, 2019). Perhaps consistency in parental behavior exists in convict cichlids but the lack of increased reproductive success commonly associated with mate fidelity results in little evolutionary benefit to remaining with the same partner. Convict cichlids do not generally display mate fidelity in natural populations (Wisenden, 1995), and so the benefits of serial monogamy likely outweigh any benefits of remaining with the same partner.

## Conclusions

Our aim was to explore the consistency in parental behavior of the convict cichlid, focusing on the influence of sex and mate fidelity. Based on the previous work in biparental cichlids and birds, we hypothesized that individuals would be consistent in their behavior and show moderate to high levels of repeatability between broods. Pairs of convict cichlids that remained together for two consecutive broods were more consistent in their parental behaviors, including time they spent near the intruder and in the nest compared to pairs that were comprised of individuals that had previously mated with other partners. Convict cichlids that remained with the same partner were also more consistent as a parental unit, maintaining their sex-specific roles of males defending aggressively against an intruder and females spending more time directly caring for young. Despite these findings of consistency in behavior when remaining with a partner, convict cichlids are rarely seen re-mating with the same individual in the field and are categorized as serially monogamous. Studies in biparental bird species suggest that high mate fidelity leads to better coordination and increased survival of offspring. Future studies of cichlids should focus on reproductive success in relation to mate fidelity to determine the ultimate and proximate mechanisms that lead to serial monogamy in natural populations.

##  Supplemental Information

10.7717/peerj.10534/supp-1 Supplemental Information 1 All behavioral data

## Additional Information and Declaration

### Data Availability

The following information was supplied regarding data availability: The raw data are available as a Supplemental File and include all parental behavioral measurements for male and female convict cichlid fishes that either remained with or were paired with a new partner. Raw data were used to compute Repeatability scores.
